# A cognitive behavioral therapy intervention to reduce fear of hypoglycemia in young adults with type 1 diabetes (FREE): study protocol for a randomized controlled trial

**DOI:** 10.1186/s13063-019-3876-4

**Published:** 2019-12-30

**Authors:** Pamela Martyn-Nemeth, Jennifer Duffecy, Laurie Quinn, Chang Park, Dan Mihailescu, Sue Penckofer

**Affiliations:** 1 Department of Biobehavioral Health Science, University of Illinois at Chicago College of Nursing, 845 South Damen Avenue (MC 802), Chicago, IL 60612 USA; 2 Department of Psychiatry, University of Illinois at Chicago, Chicago, IL USA; 3 Department of Health Systems Science, University of Illinois at Chicago College of Nursing, Chicago, IL USA; 4 Endocrinology/Diabetes and Metabolism, Department of Medicine, University of Illinois at Chicago, Chicago, IL USA; 5 Loyola University Chicago, Chicago, IL USA

**Keywords:** Fear of hypoglycemia, Type 1 diabetes, Cognitive behavioral therapy, Randomized controlled trial

## Abstract

**Background:**

In persons with type 1 diabetes (T1D), hypoglycemia is the major limiting factor in achieving optimal glycemic control. All persons with T1D are at risk for hypoglycemia (blood glucose level < 70 mg/dl), which is life-threatening and accompanied by serious physical and psychological symptoms, resulting in profound fear of hypoglycemia (FOH) and reduced quality of life. Young adults with T1D are at risk for FOH and have worse glycemic control and self-management behavior than other age groups with T1D. FOH also results in increased glycemic variability (GV). A major gap exists in how to manage FOH. Our overall objective is to reduce FOH and improve diabetes self-management, glycemic control, and GV in young adults with T1D to reduce or delay diabetes complications and improve quality of life. We aim to (1) determine the feasibility and acceptability of an eight-week cognitive behavioral therapy (CBT)-based Fear Reduction Efficacy Evaluation (FREE) intervention in young adults with T1D who experience FOH; and (2) determine the impact of the FREE intervention, compared to an attention control group, on the outcomes FOH, self-management, glycemic control (A1C), and glycemic variability (continuous glucose monitoring recordings).

**Methods/design:**

A randomized controlled trial in 50 young adults aged 18 to 35 years with T1D will be used. Eligible subjects will be randomized to the intervention program (Fear Reduction Efficacy Evaluation [FREE]) or attention control group. A one-week run-in phase is planned, with baseline measures of FOH, self-management behavior, A1C, and real-time continuous glucose monitoring recordings (RT-CGM) to calculate GV for both groups. The intervention group will participate in eight weekly individual one-hour sessions using CBT and exposure treatment for specific fears. RT-CGM and a daily FOH diary will be used as feedback cues as part of the FREE program. The attention control group will participate in eight weekly individual one-hour diabetes self-management education (DSME) sessions and wear a RT-CGM device (to measure GV only) over 8 weeks. At completion, FOH will be measured, and RT-CGM recordings will be analyzed to determine differences between the FREE and control groups.

**Discussion:**

Findings from this proposed pilot study will serve as the foundation for a larger trial to reduce FOH and improve self-management, glycemic control, and GV.

**Trial registration:**

ClinicalTrials.gov: A cognitive behavioral therapy (CBT) intervention to reduce fear of hypoglycemia in type 1 diabetes, NCT03549104. Registered June 7, 2018

## Background

In persons with type 1 diabetes (T1D), iatrogenic hypoglycemia is the major limiting factor in achieving optimal blood glucose control [1]. All persons with T1D are at risk for hypoglycemia (blood glucose level < 70 mg/dl [2]), which is life-threatening [3] and has serious physical and psychological sequelae. One psychological sequela is fear of hypoglycemia (FOH) [4, 5]. FOH can be incapacitating, causing panic [4], anxiety [6], phobic disorders [7], and greatly diminished quality of life (QOL) [8].

Despite advances in insulin therapy and glucose-sensing technology, FOH remains a critical deterrent to T1D self-management, psychological well-being, and QOL [9]. Fear is conceptualized as an emotion arising from a cognitive appraisal of a specific threat or danger [10]. Normal fear is adaptive, stimulating more vigilance and improved performance; heightened fear leads to increased anxiety and may result in a delay to action or inappropriate action [10–12]. Fear may mimic the symptoms of hypoglycemia and impair its detection, exacerbating the problem [5, 13]. At the extreme, fears can develop into anxiety disorders and phobias [7, 11, 14].

Previous negative experiences of hypoglycemia influence diabetes self-management behaviors [9, 15–20]. Diabetes self-management is defined as the knowledge, skills, and behaviors needed for diabetes self-care [21]. Insulin doses may be inappropriately reduced and diet may be modified to avoid hypoglycemia. Dietary modifications may include excessive eating, particularly more carbohydrates [22, 23] or snacking at night [19, 24]. These modifications lead to increased glycemic variability (GV; the intra-day fluctuations in blood glucose) and poor glycemic control. Registry data from the T1D Exchange Clinic Registry revealed that only 13% of young adults achieved glycemic targets [25], and, in a survey of self-management practices, 45% of young adults reported that they did not reach their glycemic goals due to FOH [9].

Young adulthood is the developmental period from age 18 to 35 years, when individuals transition to independent diabetes care, as well as establish independence, careers, family, and parenthood [26]. This is before the onset of long-term diabetes complications, when healthy behavior changes can have a critical impact on future health [27].

Over the past three decades, newer technologies have been designed to help patients with diabetes manage their treatment regimens, including continuous glucose monitoring (CGM) systems, insulin pumps, sensor-augmented pump therapy with insulin suspend features, and insulin bolus calculators. While these have improved glucose control (i.e., A1C), improved A1C has not consistently translated into reduced FOH [28–34]. Diabetes education programs typically discuss FOH but have limited strategies to manage it [4]. Glucose management and blood glucose awareness training have had variable effects on FOH [4, 35–43]. Outcomes demonstrated that, as glucose levels lower, worry levels do not consistently decrease [43]. We hypothesize that the lack of consistent and sustainable reductions in FOH has occurred because the focus of these programs has been on glycemic control, not FOH.

**Fig. 1:**
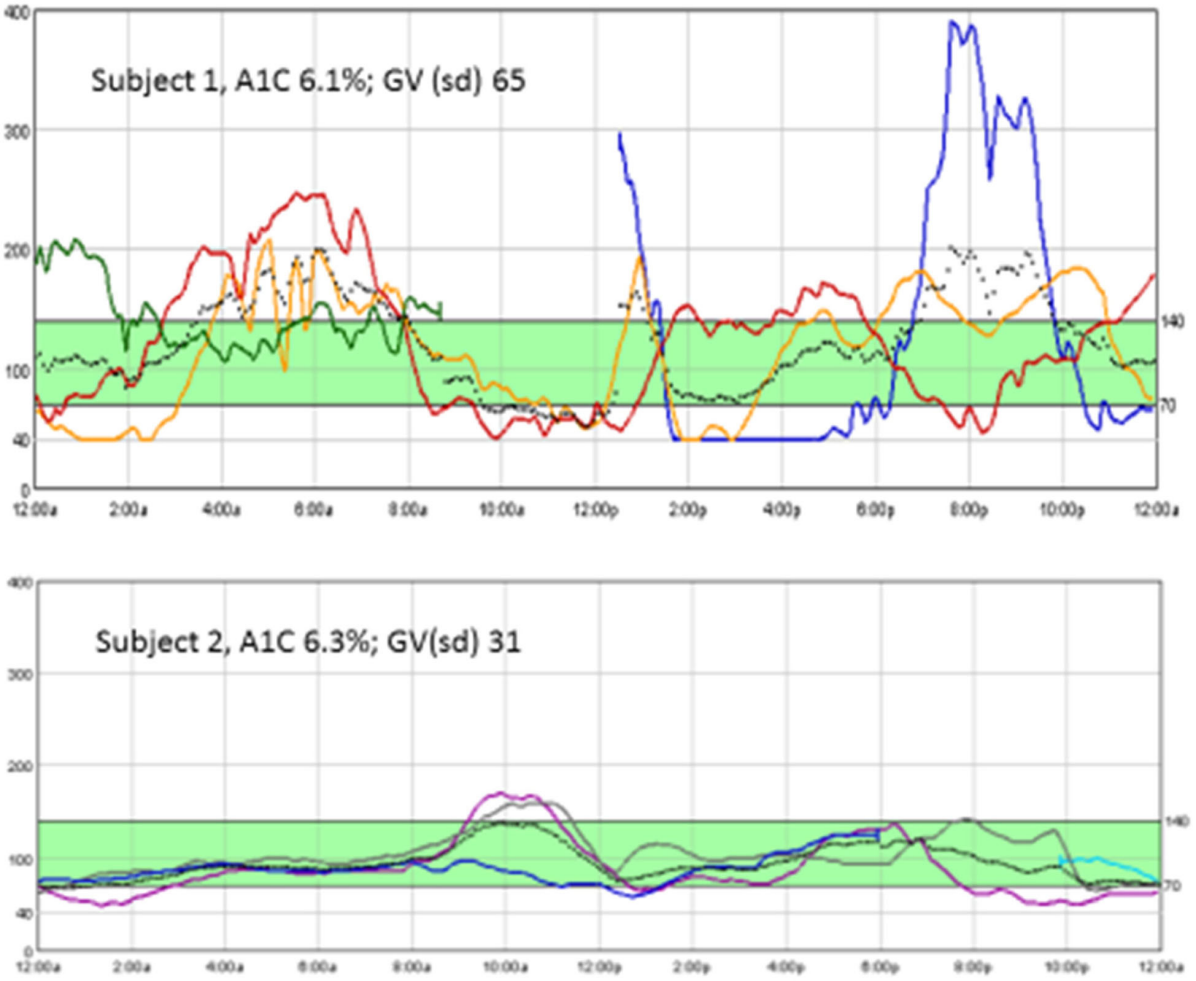
Glycemic variability

GV has been associated with more frequent episodes of hypoglycemia [44] and fluctuations between glucose extremes (i.e., hypo- to hyperglycemic), which may occur with overtreatment of a hypoglycemic episode. Evidence supports the role that GV plays in generation of oxidative stress [45], endothelial dysfunction [46], and diabetes complications in T1D [47]. GV has also been associated with increased risk for cardiovascular events [63]. Though A1C provides a biomarker for average blood glucose over a 2- to 3-month period, it does not capture daily blood glucose fluctuations (Fig. 1). Individuals may have an optimal A1C yet high GV [48]. GV is influenced by self-management behavior [49] and amenable to change with appropriate intervention.

**Fig. 2:**
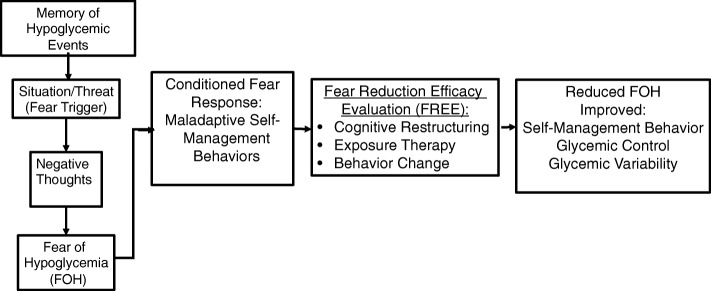
Cognitive-behavioral model of FREE

The proposed study will pilot test a cognitive behavioral therapy (CBT)-based program to decrease FOH and examine its association with GV. Heightened fear develops from memories of previous negative hypoglycemic events that create a conditioned negative response to future fear triggers [11, 50]. The conditioned fear response is reframed through cognitive restructuring of negative thoughts, regulation of emotions (FOH), and changing maladaptive behaviors (self-management behavior). Exposure therapy will be used to reduce FOH that is out of proportion to the threat through habituation to previously fearful situations (Fig. 2) [51]. Real-time continuous glucose monitor (RT-CGM) readings and fear diary review will be feedback cues to reinforce learning. The goal is not to replace standard diabetes therapy, but improve diabetes self-management [52] through reducing fear. The CBT intervention will be compared to usual diabetes education currently in place to address self-management behavior.

The specific aims for this study are:

In 50 young adults with T1D (18–35 years) [26] who experience FOH, we will:

1. Determine the feasibility and acceptability of an eight-week CBT-based Fear Reduction Efficacy Evaluation (FREE) intervention
2. Determine the impact of the FREE intervention, compared to an attention control group, on the outcomes FOH, self-management, glycemic control (A1C), and glycemic variability (RT-CGM recordings)

Primary hypothesis: Young adults with T1D participating in the FREE program will demonstrate improvement in FOH compared to the attention control group.

Secondary hypothesis: Young adults with T1D participating in the FREE program will have improved self-management, glycemic control, and glycemic variability (GV) compared to the attention control group.

## Methods/design

A randomized controlled design using parallel groups will be used to test the study hypotheses. Institutional Review Board approval was obtained (April, 2018) prior to initiating the study from the University of Illinois Office of the Protection of Human Subjects.

### Study population and recruitment

Eligible participants will include young adults (18–35 years) with T1D ≥ 1 year, receiving care from an endocrinologist, experiencing FOH (screening questionnaire) [53], who have previously attended a basic diabetes educational program. Exclusion criteria are pregnancy or breastfeeding, receiving psychotherapy for FOH, and having a co-existing chronic illness or receiving medications (excluding insulin) that may influence diabetes self-management or GV.

Participants will be recruited through two medical centers and local diabetes organizations and websites in the midwestern United States. Informed consent will be obtained from each study participant by a trained member of the study team. Those who meet study criteria will be scheduled for an appointment for the start of the one-week run-in period (week 0) and apply a RT-CGM device with instructions on its care. Participants will be randomly assigned to the FREE intervention or attention control group. During the study period, participants will continue to receive their usual diabetes care, and their health care providers will continue to care for their diabetes as they normally would.

Participants will be assigned a unique code number. A master list that links the subject identity to the data will be kept by the principal investigator (PI) and stored in a locked office separately from the data. All data will be stored and analyzed by code number. The coded data will be entered into a password-protected computer with a secure server for analysis. Paper copy data will be stored in a locked office.

Criteria for discontinuing participation for a given trial participant include: a) participant request or b) determination by the psychologist/interventionist that continuation of the study would not be appropriate due to confounding psychological issues that become evident during the study. If this occurs, a list of counseling resources will be provided.

### Sample size determination

The sample size estimate will allow examination of within-group and between-group differences from beginning to end of the intervention and to calculate effect sizes to power a future larger study. To determine the sample size to meet these goals, the mean, SD, and mean/SD of FOH levels from our pilot studies were used to estimate the expected effect size [48]. The expected estimated effect size was 0.72. Twenty-five subjects will be required in each group (treatment and control; *n* = 50 total). A 20% attrition rate is expected, based on previous psychoeducational interventions in T1D [39, 41, 43]. Thus, we will accrue 30 subjects per group, to achieve a final sample size of 25 subjects per group (*n* = 50 total).

### Randomization and masking

Randomization will be computer-generated (using REDCap) with permuted blocks in multiples of two and stratified by gender. The randomization process will be overseen by the study statistician (CP). The treatment allocation will be concealed until the time of randomization. Due to the nature of the intervention, participants and their respective interventionists will be aware of their treatment allocation. The project manager will also be aware of the treatment allocation; all other study staff will be masked. Study participants will enter questionnaire data for the primary study outcome directly into REDCap. Secondary outcomes are objectively measured, and those who will analyze these outcomes will be masked to treatment allocation until study completion. At the baseline visit, the project manager and study participant will be informed of the treatment allocation through REDCap. The project manager will then coordinate the first intervention visit with the appropriate interventionist. If an adverse event occurs, unmasking would be permissible.

### Interventions

Participants randomized to the FREE intervention group will: (1) attend eight individual weekly one-hour sessions based on principles of CBT and exposure treatment (Table 1). Treatment will target incorrect beliefs about hypoglycemia, hypervigilance to symptoms, fear of symptoms, and maladaptive behavioral responses in response to glucose levels. Participants will create a fear and avoidance hierarchy and be taught to begin approaching previously feared situations (e.g., spending time alone, reducing snacking, allowing glucose readings to reach lower safe levels, etc.) to experience habituation and the resulting decrease in anxiety. Weekly homework will be assigned to reinforce the content. (2) FREE intervention subjects will continue to wear an unblinded RT-CGM for the 8 weeks and (3) complete a daily FOH diary. RT-CGM readings and daily diaries will serve as feedback cues for glucose and FOH levels as part of the FREE program.

**Table 1:** Elements of the FREE program

Elements of the FREE Program	
Hypoglycemia and its causes	Week 1
Fear as a normal human emotion	
Effect of fear on health and health behaviors	
Safety and avoidance behaviors	
Blood glucose cues	Week 2
Introduction to CBT	
Cognitive restructuring, safety behaviors	Week 3
Introduction to progressive relaxation	
Exposure therapy: develop fear hierarchy	Week 4
Coping strategies	
Exposure practice, coping, relaxation, and cognitive restructuring	Weeks 5–7
Review techniques learned, develop a plan to maintain gains	Week 8

Participants randomized to attention control will attend eight weekly individual one-hour sessions on diabetes self-management education (Table 2). Topics follow the American Diabetes Association DSME standards [54] and will be led by a certified diabetes educator (CDE). Weekly homework will be assigned. Participants will continue to wear an unblinded RT-CGM for the 8-week session (for GV measurement *only*) but will *not* keep a diary.

**Table 2:** Elements of the attention control (DSME) program

Elements of the attention control (DSME) program	
Living with type 1 diabetes	Week 1
Using insulin safely: blood glucose monitoring	Week 2
Healthy eating: incorporating nutritional management into lifestyle	Week 3
Healthy eating: carbohydrate counting	Week 4
Being active: incorporating physical activity into lifestyle	Week 5
Healthy sleep	Week 6
Diabetes in the workplace	Week 7
Preventing complications Review and wrap-Up	Week 8

### Measures

**Fig. 3:**
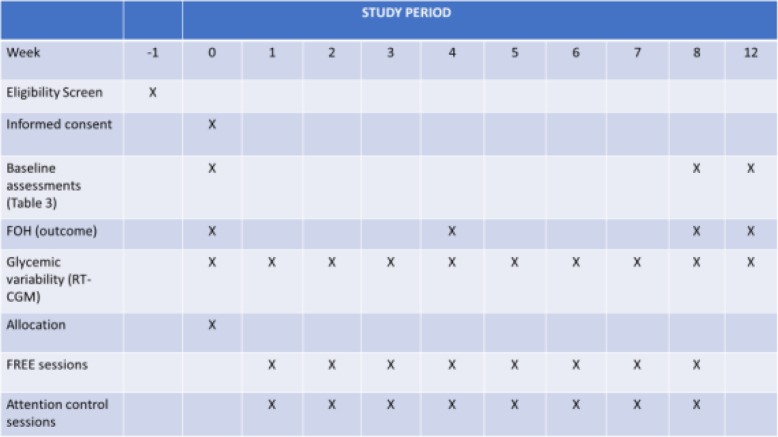
Spirit figure

Data will be collected (1) at the beginning of a one-week run-in period (week 0); (2) at week 4; (3) upon completion of the intervention (week 8); and (4) post-program (week 12). FOH, self-management, and A1C will be measured, and RT-CGM recordings will be analyzed to determine within-group and between-group differences (Fig. 3).

#### Baseline and post-intervention measures

*Self-report instruments* will be used to obtain demographic, literacy, and health information and previous history of hypoglycemia and diabetes self-management (see Table 3 for a complete list of measures). The scales chosen have strong psychometric properties and have been validated in diabetes populations. The primary outcome, FOH, will be measured using the Worry Subscale of the Hypoglycemia Fear Scale II. This 18-item, five-point Likert scale measures situation-specific worries about hypoglycemia and provides one overall score for hypoglycemic worry. The scale has strong psychometric properties (Cronbach’s alpha 0.95) [53]. Self-management will be measured with the Diabetes Self-Management Scale (Cronbach’s alpha 0.84) [56]. Convergent and construct validity are demonstrated for both scales [53, 56]. *Glycemic control* will be measured with a fingerstick for A1C using previously published methods (A1C Now®, Chek Diagnostics, Indianapolis, IN, USA) [48].

**Table 3:** Measures

Variables	Measure	Frequency
Demographic, health, and literacy information	Demographic, health questionnaire, Hypoglycemia Patient Questionnaire [2] Health Literacy Screener (Newest Vital Sign) [55]	Week 0
Aim 1		
Recruitment	Number recruited, screened, eligible, consented	Weekly
Retention	Attendance rate; completion rate	Weekly
Acceptability	Participant evaluation survey and interview (Appendix P4)	Week 8
	Advisory panel	End of study
Aim 2		
FOH	Hypoglycemia Fear Scale-II (HFS-II [52])	Weeks 0, 4, 8,12
Glycemic measures		
Glycemic control	A1C (A1C Now®)	Weeks 0, 8, 12
Glycemic variability	RT-CGM (Dexcom®): daily glucose standard deviation (GlucSD), continuous net glycemic action (CONGA), coefficient of variation (CV%), interquartile range (IQR), time spent in hypo- and hyperglycemia and time in range	Weeks 0–8
Diabetes self-management	Diabetes Self-Management Questionnaire [54]	Weeks 0, 8, 12

#### Glycemic variability

Subcutaneous interstitial glucose levels will be monitored using Dexcom G-series continuous glucose monitoring systems® (San Diego, CA). To analyze GV, the frequency and time spent in hypo- and hyperglycemia and time in range will be calculated (percentage and minutes; < 70 and > 180 mg/dL). GV will be determined by calculating the daily glucose standard deviation (GlucSD), continuous net glycemic action (CONGA), coefficient of variation (CV), and interquartile range (IQR) [57, 58].

#### Related variables

Self-efficacy, anxiety, diabetes distress, depressive mood, and quality of life will be measured with validated instruments (Self-Efficacy for Diabetes Scale [59], General Anxiety Disorder-7 Item [GAD-7] [60], Diabetes Distress Scale [61], Center for Epidemiological Studies Depression Scale [CES-D] [62], and Ferrans and Powers Quality of Life Index-Diabetes [55], respectively, at weeks 0, 8, and 12).

### Data safety monitoring

A data safety monitoring committee (DSMC) will be formed to evaluate accumulated data for participation safety, study conduct, and progress. The DSMC will be comprised of an interdisciplinary team of professors from the University of Illinois at Chicago and will be independent of the study sponsor. The DSMC will submit an annual report to the study team, University Institutional Review Board, and the study sponsor.

### Statistical analysis

The statistical estimation method for this study is a mixed-effects model with repeated measures (SPSS).

Aim 1: Feasibility will be evaluated by assessing recruitment, retention, and participant evaluation. Records will be kept of the number of recruited, screened, eligible, and consented subjects. Retention will be evaluated by weekly attendance (percentage session attendance, program completion rates). Acceptability will be determined through participant evaluation (written evaluation and interview at program completion and a convened advisory group of previous participants). As part of feasibility, we will also track the number of RT-CGM sensor failures, placement sites, time to failure, and adverse sensor site problems.

Aim 2: We will evaluate the effects of within-group and between-group differences from baseline (week 0) to program completion (week 8) and post program (week 12) on the outcomes FOH, self-management, glycemic control, and GV, using an intent-to-treat approach. To address sex as a biologic variable, sex differences in primary and secondary outcomes will be explored. Diabetes duration and depressive mood will also be statistically controlled.

Hypothesis 1: FOH will be reduced. Within-group and between-group differences in HFS worry score from baseline to study completion and post program will be compared using a mixed-effects model.

Hypothesis 2a: Diabetes self-management will be improved. Within- and between-group differences in the Diabetes Self-Management Scale score from baseline to study completion and post-program will be compared using a mixed-effects model.

Hypothesis 2b: Glycemic control. Within- and between-group differences in A1C from baseline to study completion and post-program will be compared using a mixed-effects model.

Hypothesis 2c: GV will decrease. GV will be determined through GlucSD, CONGA, CV%, and IQR, as well as daily time spent in hypo- or hyperglycemia. Within- and between-group differences will be calculated from daily RT-CGM recordings using a mixed-effects model.

#### Management of missing data

Missing values will be analyzed to better understand the characteristics of missing patterns. Once the missing patterns are understood, an imputation method will be determined if appropriate. As this is a pilot study, missing value information will be used to plan the next stage and larger study.

### Participant retention strategy

Both FREE intervention and attention control groups will (1) have weekly sessions scheduled at a time convenient for the participants; (2) wear a RT-CGM; (3) receive appointment reminders; (4) be compensated at weeks 4, 8, and 12; and (5) receive a personalized folder with copies of their CGM recordings at study’s end. Weekly sessions for both the FREE and attention control groups will include topics of interest and value to the study population. Use of RT-CGM technology is highly desirable for many young adults with T1D and served as both an incentive and retention factor in our previous studies [48].

### Intervention fidelity

To maintain treatment fidelity, both FREE and attention control sessions will follow a manualized protocol. Also, sessions will be audio-recorded and reviewed for fidelity to the treatment protocol by study staff.

## Discussion

This study protocol directly focuses on FOH reduction. This study will generate information for a larger clinical trial to test the effectiveness of the FREE intervention compared to diabetes management education to reduce FOH, improve self-management behavior, and improve glycemic control and GV. If effective, this intervention will serve as an important adjunct to diabetes care in young adults with T1D, reduce the development of diabetes complications, and improve quality of life.

### Trial status

This study is in the recruitment phase (protocol version 7, 5/17/2019). Recruitment began November 2018. The first participant was enrolled January 2019. Recruitment is expected to be completed by June 2020.

## Supplementary information

**Additional file 1.** SPIRIT 2013 checklist

## Data Availability

Not applicable at this time.

## Abbreviations

A1C: Glycated hemoglobin
CBT: Cognitive behavioral therapy
CES-D: Center for Epidemiological Studies Depression Scale
CGM: Continuous glucose monitoring
CONGA: Continuous net glycemic action
CV: Coefficient of variation
FOH: Fear of hypoglycemia
FREE: Fear reduction efficacy evaluation
GAD: General Anxiety Disorder Scale
GlucSD: Glucose standard deviation
GV: Glycemic variability
HFS-II: Hypoglycemia Fear Scale
IQR: Interquartile range
QOL: Quality of life
REDCap: Research Electronic Data Capture
RT-CGM: Real-time continuous glucose monitoring
T1D: Type 1 diabetes
