# Supplementary material for: A cognitive behavioral therapy intervention to reduce fear of hypoglycemia in young adults with type 1 diabetes (FREE): study protocol for a randomized controlled trial
Source: Trials. 2019 Dec 30;20:796. doi: 10.1186/s13063-019-3876-4 (PMC6938021; doi:10.1186/s13063-019-3876-4)
Supplement: Supplementary file 1 — Additional file 1. SPIRIT 2013 checklist [file 13063_2019_3876_MOESM1_ESM.doc]

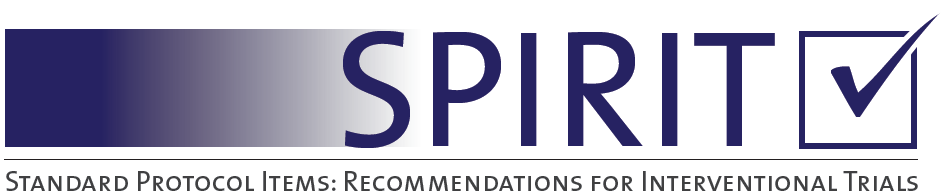


A Cognitive Behavioral Therapy Intervention to Reduce Fear of Hypoglycemia in Young Adults with Type 1 Diabetes (FREE): Study Protocol for a Randomized Controlled Trial

SPIRIT 2013 Checklist: Recommended items to address in a clinical trial protocol and related documents*

| Section/item | Item No | Description | Addressed on page number |
| --- | --- | --- | --- |
| **Administrative information** | | |  |
| Title | 1 | Descriptive title identifying the study design, population, interventions, and, if applicable, trial acronym | Y-Title page |
| Trial registration | 2a | Trial identifier and registry name. If not yet registered, name of intended registry | Y-page 3, NCT03549104 |
|  | 2b | All items from the World Health Organization Trial Registration Data Set | Please refer to item 2a. |
| Protocol version | 3 | Date and version identifier | 5/17/2019, Ver 7 |
| Funding | 4 | Sources and types of financial, material, and other support | Funding agency: NIH-NIDDK: R21DK116146 (page 13) |
| Roles and responsibilities | 5a | Names, affiliations, and roles of protocol contributors | Y-Title page and page 13 |
|  | 5b | Name and contact information for the trial sponsor | Y- page 13 |
|  | 5c | Role of study sponsor and funders, if any, in study design; collection, management, analysis, and interpretation of data; writing of the report; and the decision to submit the report for publication, including whether they will have ultimate authority over any of these activities | The sponsor played no part in study design; collection, management, and analysis plan for the data; writing of this report; and the decision to submit the report for publication (page 14). |
|  | 5d | Composition, roles, and responsibilities of the coordinating centre, steering committee, endpoint adjudication committee, data management team, and other individuals or groups overseeing the trial, if applicable (see Item 21a for data monitoring committee) | The trial steering committee is comprised of the protocol authors. The data safety monitoring committee reviews the study annually. |
| Introduction |  |  |  |
| Background and rationale | 6a | Description of research question and justification for undertaking the trial, including summary of relevant studies (published and unpublished) examining benefits and harms for each intervention | Y-pages 2-3 |
|  | 6b | Explanation for choice of comparators | Y- pages 4-6 |
| Objectives | 7 | Specific objectives or hypotheses | Y- page 6 |
| Trial design | 8 | Description of trial design including type of trial (eg, parallel group, crossover, factorial, single group), allocation ratio, and framework (eg, superiority, equivalence, noninferiority, exploratory) | Y – pages 6-8 |
| Methods: Participants, interventions, and outcomes | | |  |
| Study setting | 9 | Description of study settings (eg, community clinic, academic hospital) and list of countries where data will be collected. Reference to where list of study sites can be obtained | Y – page 7 |
| Eligibility criteria | 10 | Inclusion and exclusion criteria for participants. If applicable, eligibility criteria for study centres and individuals who will perform the interventions (eg, surgeons, psychotherapists) | Y – page 7 |
| Interventions | 11a | Interventions for each group with sufficient detail to allow replication, including how and when they will be administered | Y – pages 8-9 |
|  | 11b | Criteria for discontinuing or modifying allocated interventions for a given trial participant (eg, drug dose change in response to harms, participant request, or improving/worsening disease) | 1. Criteria for discontinuation includes: a.) participant request ; b.) If the psychologist interventionist determines that continuation in the FREE program would not be appropriate due to confounding psychological issues that become evident during the study, continuation in the study may be stopped and a list of counseling agencies provided. See page 7. |
|  | 11c | Strategies to improve adherence to intervention protocols, and any procedures for monitoring adherence (eg, drug tablet return, laboratory tests) | Y – pages 8-9. Intervention fidelity plan p. 12 |
|  | 11d | Relevant concomitant care and interventions that are permitted or prohibited during the trial | Participants will receive their usual care from their diabetes team. See page 7. |
| Outcomes | 12 | Primary, secondary, and other outcomes, including the specific measurement variable (eg, systolic blood pressure), analysis metric (eg, change from baseline, final value, time to event), method of aggregation (eg, median, proportion), and time point for each outcome. Explanation of the clinical relevance of chosen efficacy and harm outcomes is strongly recommended | Y – Measures: pages 9-10; Outcomes: Statistical Analysis, pages 10-11. |
| Participant timeline | 13 | Time schedule of enrollment, interventions (including any run-ins and washouts), assessments, and visits for participants. A schematic diagram is highly recommended (see Figure) | Y – see Spirit diagram, page 9. |
| Sample size | 14 | Estimated number of participants needed to achieve study objectives and how it was determined, including clinical and statistical assumptions supporting any sample size calculations | Y – see Sample Size Determination, page 7 |
| Recruitment | 15 | Strategies for achieving adequate participant enrolment to reach target sample size | Y – see page 7 |
| **Methods: Assignment of interventions (for controlled trials)** | | |  |
| Allocation: |  |  |  |
| Sequence generation | 16a | Method of generating the allocation sequence (eg, computer-generated random numbers), and list of any factors for stratification. To reduce predictability of a random sequence, details of any planned restriction (eg, blocking) should be provided in a separate document that is unavailable to those who enrol participants or assign interventions | Y – See Randomization and Masking, pages 7-8 |
| Allocation concealment mechanism | 16b | Mechanism of implementing the allocation sequence (eg, central telephone; sequentially numbered, opaque, sealed envelopes), describing any steps to conceal the sequence until interventions are assigned | Y – See Randomization and Masking, pages 7-8 |
| Implementation | 16c | Who will generate the allocation sequence, who will enroll participants, and who will assign participants to interventions | See pages 7-8 |
| Blinding (masking) | 17a | Who will be blinded after assignment to interventions (eg, trial participants, care providers, outcome assessors, data analysts), and how | See pages 7-8 |
|  | 17b | If blinded, circumstances under which unblinding is permissible, and procedure for revealing a participant’s allocated intervention during the trial | If an adverse event occurred, unmasking would be permissible. The project director is unmasked and would notify the PI (who is masked) of the adverse event. Page 8. |
| **Methods: Data collection, management, and analysis** | | |  |
| Data collection methods | 18a | Plans for assessment and collection of outcome, baseline, and other trial data, including any related processes to promote data quality (eg, duplicate measurements, training of assessors) and a description of study instruments (eg, questionnaires, laboratory tests) along with their reliability and validity, if known. Reference to where data collection forms can be found, if not in the protocol | Questionnaire data is collected through REDCap and is evaluated for completeness at the time of data collection. Paper copies are stored in a master file if needed. For a description of study instruments, see pages 9-10 and Table 3. |
|  | 18b | Plans to promote participant retention and complete follow-up, including list of any outcome data to be collected for participants who discontinue or deviate from intervention protocols | See page 11 for participant retention plan. Data will be analysed with an intention-to-treat methodology. |
| Data management | 19 | Plans for data entry, coding, security, and storage, including any related processes to promote data quality (eg, double data entry; range checks for data values). Reference to where details of data management procedures can be found, if not in the protocol | Data are entered into REDCap, with a second copy stored in a HIPAA-protected Box folder maintained by the university. Data files will be checked in duplicate and values evaluated for accuracy. Details of data management procedures are described here and located in the study manual and IRB Initial Review Application. |
| Statistical methods | 20a | Statistical methods for analysing primary and secondary outcomes. Reference to where other details of the statistical analysis plan can be found, if not in the protocol | See Statistical Analysis: pages 10- 11. |
|  | 20b | Methods for any additional analyses (eg, subgroup and adjusted analyses) | See pages 10-11. |
|  | 20c | Definition of analysis population relating to protocol non-adherence (eg, as randomised analysis), and any statistical methods to handle missing data (eg, multiple imputation) | Because no previous studies for this population were available for the missing patterns, missing value analysis will help to understand the unique characteristics of missing patterns and causes of it. Once the missing patterns are understood, an imputation method will be determined including multiple imputation method. As this is a pilot and feasibility study, missing value information will be used to plan the next stage and larger study. See Management of Missing Data, page 11. |
| **Methods: Monitoring** | | |  |
| Data monitoring | 21a | Composition of data monitoring committee (DMC); summary of its role and reporting structure; statement of whether it is independent from the sponsor and competing interests; and reference to where further details about its charter can be found, if not in the protocol. Alternatively, an explanation of why a DMC is not needed | DSC is composed of Professors of the University of Illinois at Chicago: one endocrinologist, one psychologist, and one nurse. The role of the DSC is to periodically review and evaluate the accumulated study data for participant safety, study conduct and progress and 2) make recommendations to the study team concerning the continuation, modification, or termination of the trial. They are independent from the sponsor. Minutes of meetings are submitted to the Institutional Review Board of the University and to the NIH. See Data Safety Monitoring, page 10. |
|  | 21b | Description of any interim analyses and stopping guidelines, including who will have access to these interim results and make the final decision to terminate the trial | Interim analyses are conducted by the DSMC (annually and more frequently if needed); and annual report to the NIH. See page Data Safety Monitoring, page 10. |
| Harms | 22 | Plans for collecting, assessing, reporting, and managing solicited and spontaneously reported adverse events and other unintended effects of trial interventions or trial conduct | All unintended or adverse effects are reported to the University Institutional Review Board per policy. Also see Data Safety Monitoring, page 10. |
| Auditing | 23 | Frequency and procedures for auditing trial conduct, if any, and whether the process will be independent from investigators and the sponsor | As described above. |
| Ethics and dissemination | | |  |
| Research ethics approval | 24 | Plans for seeking research ethics committee/institutional review board (REC/IRB) approval | IRB approval was obtained prior to study initiation. See Methods/Design, page 6. |
| Protocol amendments | 25 | Plans for communicating important protocol modifications (eg, changes to eligibility criteria, outcomes, analyses) to relevant parties (eg, investigators, REC/IRBs, trial participants, trial registries, journals, regulators) | All protocol amendments are submitted to the University IRB for approval. Sponsor approval is obtained for major modifications and trials registry is updated as appropriate. |
| Consent or assent | 26a | Who will obtain informed consent or assent from potential trial participants or authorised surrogates, and how (see Item 32) | Informed consent will be obtained by the PI or key research personnel on the study team. See Population and Recruitment, page 7. |
|  | 26b | Additional consent provisions for collection and use of participant data and biological specimens in ancillary studies, if applicable | Participants will be asked for permission for the research team to share relevant data with people from the University taking part in the research or from regulatory authorities where relevant (Consent page 8). Participants who choose to withdraw from the study may request that future health information not be used (consent page 9). This trial does not involve collecting biological specimens for storage. |
| Confidentiality | 27 | How personal information about potential and enrolled participants will be collected, shared, and maintained in order to protect confidentiality before, during, and after the trial | Participants will be assigned a unique code number. A master list that links the subject identity to the data will be kept by the principal investigator (PI) and stored in a locked office separately from the data. **Data storage**: All data will be stored and analyzed by code number. The coded data will be entered into a password-protected computer with a secure server for analysis. Paper copy data will be stored in a locked office as described in IRB Initial Application. |
| Declaration of interests | 28 | Financial and other competing interests for principal investigators for the overall trial and each study site | All authors declare that they have no conflicts or competing interests.This study is funded by the National Institutes of Health, NIDDK (R21DK116146). |
| Access to data | 29 | Statement of who will have access to the final trial dataset, and disclosure of contractual agreements that limit such access for investigators | The PI will have access to the final trial dataset. Any data required to support the protocol can be supplied upon request. |
| Ancillary and post-trial care | 30 | Provisions, if any, for ancillary and post-trial care, and for compensation to those who suffer harm from trial participation | Described in consent. (Version 8, 5-17-19). |
| Dissemination policy | 31a | Plans for investigators and sponsor to communicate trial results to participants, healthcare professionals, the public, and other relevant groups (eg, via publication, reporting in results databases, or other data sharing arrangements), including any publication restrictions | The dissemination plan includes: 1.) final study report submitted to the sponsor, 2.) manuscript submission, 3.) communication of findings to study participants, and 4.) reporting of results in ClinicalTrials.gov. |
|  | 31b | Authorship eligibility guidelines and any intended use of professional writers | No professional writers will be used. All named authors adhere to the authorship guidelines of *Trials,* have agreed to publication and have contributed to writing the manuscript as detailed on pp 13-14. |
|  | 31c | Plans, if any, for granting public access to the full protocol, participant-level dataset, and statistical code | The datasets analyzed during the current study may be available from the corresponding author on reasonable request. |
| Appendices |  |  |  |
| Informed consent materials | 32 | Model consent form and other related documentation given to participants and authorised surrogates | Version 8, 5-17-19 |
| Biological specimens | 33 | Plans for collection, laboratory evaluation, and storage of biological specimens for genetic or molecular analysis in the current trial and for future use in ancillary studies, if applicable | This trial does not involve collecting biological specimens for storage. |

*It is strongly recommended that this checklist be read in conjunction with the SPIRIT 2013 Explanation & Elaboration for important clarification on the items. Amendments to the protocol should be tracked and dated. The SPIRIT checklist is copyrighted by the SPIRIT Group under the Creative Commons “[Attribution-NonCommercial-NoDerivs 3.0 Unported](http://www.creativecommons.org/licenses/by-nc-nd/3.0/)” license.
